# Palmistry

**Published:** 1889-08-03

**Authors:** 


### PALMISTRY.

How should we regard palmistry—as a folly, a fanciful superstition suitable for the graceful unreality of the fancy bazaar, or as a language of Nature, a hieroglyph in which she inscribes her opinion of her own work? The two theories are recorded side by side in the last number of the *Universal Review*, where Mr. W. L. Courtney proves that the theory of palmistry is unscientific and absurd, and Mr. Edwin Ellis agrees as to its empirical character, but adds, in effect, "it's true for all that." Both gentlemen are right, in all probability. The rules of palmistry are of the most arbitrary nature. A "mount," or cushion under the first finger, indicates the influence of the planet Jupiter, we learn in the literature of the subject. Now, the calling of the large planet with its attendant moons by the name of the Monarch of Olympus was a purely arbitrary matter, and gave no more imperial power or influence to the distant star than giving the name of Victoria to a girl child secures to her a claim to the throne of England. The mount on the hand was also named in arbitrary fashion; has no real connection with the planet, nor with Jupiter himself, assuming even that the god is more than a myth. So far, so good. Any attempt to connect the mount of Jupiter with the qualities supposed to characterise Jupiter is absurd and unscientific. But if that mount occurs invariably in the hand of ambitious people, and is larger in proportion to their ambition, are we not justified in regarding it as in some way a sign of that quality? "Arbitrary" and "empirical" are good words with which to label anything that cannot be explained; but these arbitrary and empirical ideas are often the result of observations none the less keen and reliable that they are not published regularly in a scientific journal. Palmistry, we are willing to admit, cannot be explained on a scientific basis—that is, on any scientific basis yet discovered—and those who believe in it usually make out a bad case for themselves when they try. On the other hand, they are pefectly safe when they say—having seen such a sign on the hand accompanying a certain quality in the character—"I believe that the sign may be taken as an indicator of the quality."

We speak of character, not fortune, except in so far as fortune depends on character. Nature cannot tell you if your uncle will leave his money to you or to the Home for Friendless Parrots, but it will indicate clearly enough whether you have strength and will to make your own way in the world. We admit the general indications of physiognomy; those of palmistry, or more properly cheirognomy, for indications of character are given by the general formation of the hand as well as by the lines of the palm. Flat-tipped fingers usually go with cool common-sense; long-pointed tips belong to the idealists. The devolpment of the palm will give indications of success or failure; a broad, strong palm will dominate even slim, weak fingers, and give a general strength to the character, even when the fingers tell at what cost to natural tenderness and a desire to yield to the wishes of others that strength is gained.

We may seem, perhaps, to be dealing too seriously with a pastime of giddy or love-sick girls who want to be assured that they will soon be married to a rich and handsome lover. But, indeed, palmistry has little to do with these incidents, in spite of the predictions of the gipsy whose hand is duly crossed with silver. But the shape and markings of the hand indicate constitution, as any doctor will tell. On constitution largely depends temperament, and temperament is the foundation of character. Thus, without calling in the aid of the planets or throwing overboard entirely modern habits of thought, it may be possible to find a satisfactory and not wholly unscientific basis for the art of palmistry.

